# Reshaping the Preterm Heart: Shifting Cardiac Renin-Angiotensin System Towards Cardioprotection in Rats Exposed to Neonatal High-Oxygen Stress

**DOI:** 10.1161/HYPERTENSIONAHA.122.19115

**Published:** 2022-05-19

**Authors:** Mariane Bertagnolli, Daniela R. Dartora, Pablo Lamata, Ernesto Zacur, Thuy-An Mai-Vo, Ying He, Léonie Beauchamp, Adam J. Lewandowski, Anik Cloutier, Megan R. Sutherland, Robson A.S. Santos, Anne Monique Nuyt

**Affiliations:** Sainte-Justine University Hospital Research Center, Université de Montréal, Canada (M.B., D.R.D., T.-A.M.-V., Y.H., L.B., A.C., M.R.S., A.M.N.).; Research Center of the Hospital Sacré-Coeur, CIUSSS Nord-de-l’Île-de-Montréal, Canada (M.B.).; School of Physical and Occupational Therapy, Faculty of Medicine, McGill University, Montréal, Canada (M.B.).; Instituto de Cardiologia de Porto Alegre, Fundação Universitária de Cardiologia, Brazil (D.R.D.).; Department of Biomedical Engineering, Division of Imaging Sciences and Biomedical Engineering, King’s College London, United Kingdom (P.L., E.Z.).; Oxford Cardiovascular Clinical Research Facility, Division of Cardiovascular Medicine, Radcliffe Department of Medicine, University of Oxford, United Kingdom (A.J.L.).; Monash Biomedicine Discovery Institute, Monash University, Clayton, Victoria, Australia (M.R.S.).; Department of Physiology, Instituto Nacional de Ciência e Tecnologia – Nanobiofar, Universidade Federal de Minas Gerais, Belo Horizonte, Brazil (R.A.S.S.).

**Keywords:** angiotensin-converting enzyme, cardiomyopathies, cyclodextrins, echocardiography, premature birth, renin-angiotensin system

## Abstract

**Background:**

Approximately 10% of infants are born preterm. Preterm birth leads to short and long-term changes in cardiac shape and function. By using a rat model of neonatal high-oxygen (80%O_2_) exposure, mimicking the premature hyperoxic transition to the extrauterine environment, we revealed a major role of the renin-angiotensin system peptide Angio II (angiotensin II) and its receptor AT1 (angiotensin receptor type 1) on neonatal O_2_-induced cardiomyopathy. Here, we tested whether treatment with either orally active compounds of the peptides Angio-(1–7) or alamandine included in cyclodextrin could prevent postnatal cardiac remodeling and the programming of cardiomyopathy induced by neonatal high-O_2_ exposure.

**Methods:**

Sprague-Dawley pups were exposed to room air or 80% O_2_ from postnatal day 3 (P3) to P10. Neonatal rats were treated orally from P3 to P10 and assessed at P10 and P28. Left ventricular (LV) shapes were characterized by tridimensional computational atlases of ultrasound images in addition to histomorphometry.

**Results:**

At P10, high O_2_-exposed rats presented a smaller, globular and hypertrophied LV shape versus controls. Treatment with cyclodextrin–Angio-(1–7) significantly improved LV function in the O_2_-exposed neonatal rats and slightly changed LV shape. Cyclodextrin-alamandine and cyclodextrin–Angio-(1–7) treatments similarly reduced hypertrophy at P10 as well as LV remodeling and dysfunction at P28. Both treatments upregulated cardiac angiotensin-converting enzyme 2 in O_2_-exposed rats at P10 and P28.

**Conclusions:**

Our findings demonstrate LV remodeling changes induced by O_2_-stress and the potential benefits of treatments targeting the cardioprotective renin-angiotensin system axis, supporting the neonatal period as an important window for interventions aiming at preventing cardiomyopathy in people born preterm.

**Novelty and Relevance**

**What Is New?**

Orally active compounds of the peptides angiotensin-(1–7) or alamandine prevented postnatal cardiomyocyte hypertrophy and the programming of cardiomyopathy in rats exposed to neonatal high-O_2_.

**What Is Relevant?**

Neonatal rats exposed to high-O_2_ have different cardiac remodeling patterns associated with a higher predisposition to develop cardiomyopathy into adulthood, which were prevented by both treatments and more significantly modified by angiotensin-(1–7).
Both treatments stimulated the upregulation of cardiac ACE (angiotensin-converting enzyme) 2, which may balance renin-angiotensin system and prevent the deleterious effects of angiotensin II/AT1 (angiotensin receptor type 1) on heart remodeling and programming of cardiomyopathy in this model.

**Clinical/Pathophysiological Implications?**

Our findings demonstrate the potential benefits of treatments targeting the cardioprotective renin-angiotensin system axis and support the neonatal period as an important window for interventions aiming at preventing cardiomyopathy in people born preterm.

Globally, ≈10% of all infants are born less than 37 gestational weeks, and 1% are extremely preterm (<28 weeks).^1^ Over the past few decades, the rapid translation of scientific findings in this field into clinical practice has stimulated significant improvements in perinatal care. As a direct consequence, nearly 90% of these infants now survive, including those born the most premature. As the first generation of survivors reaches adulthood, long-term health consequences have started to be revealed.^2–4^

Although the causes and risk factors of preterm birth are multiple, common clinical cardiovascular implications beyond the neonatal period are now shown as early as childhood.^2^ Changes in heart shape and function are evidence of postnatal cardiac remodeling, which is one of the first signs of cardiovascular adaptations to preterm birth stress.^5–8^ The abrupt hemodynamic shift to a circulatory overload, plus systemic oxidative stress triggered by the early exposure to higher oxygen (O_2_) concentrations in the extrauterine environment, are conditions imposed on all preterm newborns at a time when the heart is undergoing an accelerated growth phase.^9–11^ As a result, the hearts of people born preterm are reshaped, with ventricular changes also evident as they grow older.^5,8^ In addition, they show a reduced myocardial efficiency and ventricular systolic dysfunction during physiological exercise stress.^12^

We have previously reported similar findings in a well-established rat model of prematurity-related complications caused by transient neonatal high-O_2_ exposure (80% O_2_) from 3 to 10 days of life,^13–16^ an age at which cardiomyocyte proliferation is ongoing,^17^ mimicking the oxidant stress burst present during preterm birth. By using this model, we first described the progression of left ventricular (LV) remodeling and dysfunction in male juvenile (28 days old) rats before any blood pressure changes, which only occur after 6 to 7 weeks in this model.^18,19^ Furthermore, we demonstrated that aging and hemodynamic overload predisposed O_2_-exposed rats to a rapid transition to heart failure.^18^ Hence, clinical and experimental findings underline an inefficient myocardium adaption, being a relevant process contributing to increased risk of heart disease, which has been consistently observed in the preterm-born population.^2,4^ However, there is still a knowledge gap on key mechanisms promoting adverse myocardium adaptations and, most importantly, on therapies capable of preventing adverse cardiac remodeling and diseases in preterm-born individuals.

The renin-angiotensin system (RAS) has been shown by us and others to exert an intriguing dueling role in preterm birth, particularly in neonatal heart development.^18,20,21^ RAS consists of 2 antagonistic branches: one prohypertensive and fibrogenic axis composed of ACE (angiotensin-converting enzyme), Angio II (angiotensin II), and its receptors AT1 (angiotensin receptor type 1) and AT2 (angiotensin receptor type 2), known as the ACE/Angio II/AT1-AT2 axis, and the counter regulatory axis with antifibrogenic and cardioprotective properties composed of ACE2, Angio-(1–7) and its receptor Mas, known as the ACE2/Angio-(1–7)/Mas axis. In addition, a newly discovered branch of the system has been added and is formed by alamandine and its receptor MrgD, which is also associated with cardioprotective effects.^22^ At birth and early postnatal days (P), ACE, the enzyme cleaving Angio I to form Angio II, and both Angio II receptors AT1 and AT2, are physiologically upregulated in hearts of neonatal rats and further exacerbated in those exposed to hyperoxia.^20^ These main components of the prohypertensive ACE/Angio II/ AT1 axis remain upregulated as they grow older and significantly contribute to the establishment of LV remodeling and dysfunction in this model, which can be prevented by short-term neonatal treatment with losartan.^20^ Despite being effective at preventing the developmental programming of cardiac dysfunction in our rat model, clinically, the use of losartan and other RAS blockers is contraindicated in newborns due to the high risk of cardiac and renal malformations.^23,24^ Therefore, in this study, we hypothesized that alternative RAS-related therapies that could potentially shift the RAS towards its cardioprotective axis, without blocking the physiological role of Angio II and AT1, could prevent postnatal cardiac remodeling and the early programming of cardiomyopathy induced by neonatal high O_2_ exposure.

Here, we aim to describe the effects of neonatal treatments with orally active compounds of Angio-(1–7) or alamandine included in β-cyclodextrins on neonatal heart’s shape, remodeling, and function associated with molecular changes in cardiac RAS profile, and on the long-term programming of ventricular dysfunction induced by neonatal high-O_2_ exposure. To reach this goal, in addition to the functional and histomorphological cardiac assessments, we developed a noninvasive method of ultrasound imaging of neonatal rats to quantitatively capture in vivo 3-dimensional (3D) geometric ventricular changes caused by neonatal high-O_2_ stress and treatments.

## Methods

The authors declare that all supporting data are available within the article and its Supplemental Material.

### Animal Model

Experiments were performed on 36 Sprague-Dawley litters each culled to n=12 (50% male) pups. Pups were kept in an oxycycler ProOx P110 (Biosherix, Lacona, NY) at 80% O_2_ concentration (high O_2_) or room air (control) from P3 to P10, as previously described.^18,19^ To avoid maternal morbidity associated with O_2_-toxicity, mothers from the room air and high O_2_-exposed groups were interchanged every 12 hours, which was previously shown to not affect pup growth or blood pressure levels in this model.^19^ Each litter was divided into 3 experimental treatment groups: vehicle cyclodextrin (30 μg/kg per day), cyclodextrin–Angio-(1–7) or cyclodextrin-alamandine oral formulation (inclusion compounds: 74 μg/kg per day [30 μg/kg per day of cyclodextrin+44 μg/kg per day of included peptides])^25^ from P3 to P10, administered daily by gavage. Different litters were used to assess or carry the assessments at P10 and P28. No more than 2 pups per litter were used per treatment group in each experiment. P28 was chosen to study the effect of therapies on cardiac changes as previously reported by us and before blood pressure changes and overload in this model.^18,26^ Only male rats were used because our data showed no significant cardiovascular alterations in female offspring at P28 before blood pressure changes in this model (Yzydorczyk et al^19^ and Figure S1). All experimental procedures were approved by the Animal Ethics Committee of the Sainte-Justine University Hospital Research Center and followed the guidelines of the Canadian Council on Animal Care and the National Institutes of Health Guide for the Care and Use of Laboratory Animals.

### Comprehensive Analysis of the Neonatal LV Anatomy by Echocardiography and 3D Computational Models

The method to characterize in vivo LV remodeling was developed by combining echocardiography and computational techniques. It was performed on 24 neonatal rats at P10, immediately after O_2_ exposure and treatments (Figure S2A). To obtain 3D images, VEVO3100 ultrasound system (VisualSonics) and a 30 MHz probe were used. 3D ventricular volumes were recorded using the 3D feature of VEVO3100 system and automated mechanical arm (Figure S2B) by recording ≈100 sequential LV images in the short-axis view, with a distance of ≈0.1 mm between each image. Segmentation of the LV myocardium was then performed semiautomatically using the Medical Imaging Interaction Toolkit software (http://www.mitk.org/wiki/MITK). 3D meshes using cubic interpolation basis functions were then fitted to the segmentation masks using image registration and an idealized LV template, adopting the methods described by Lamata et al.^27^ Meshes were then aligned by their center of mass and orientation, and an average anatomy was obtained, as illustrated in the atlas presented in Figure S2C through S2D. A principal component analysis (PCA) was then used to analyze the differences of meshes to the average, identifying the 23 linear PCA modes of geometric variation. The amount of variance explained by the 2 main modes and the cumulative variance explained by groups of modes are shown in Figure S2E. In addition, Figure S2F illustrates the 2 first modes of variation that explained 33.64% of the variance observed in all cases. As a result of this analysis, the LV anatomy of each of the 24 cases is described by 2 coefficients corresponding to the first 2 PCA modes. Each mode was compared between experimental groups to detect shape changes caused by O_2_-exposure and drug treatments. In addition, conventional LV bidimensional short-axis M-mode and mitral valve flow Doppler imaging, myocardium strain, and Tissue Doppler analyses were performed. LV mass was calculated by LV mass=1.053×([LV internal diameter in diastole+LV posterior wall thickness in diastole+intraventricular septum thickness in diastole]^3^–LV internal diameter in diastole^3^).

### Additional Experimental and Molecular Procedures

We additionally performed echocardiography in juvenile rats at P28 (see Supplemental Material). Hearts and kidneys were collected after euthanasia immediately following echocardiography imaging. Hearts were washed in 100 mmol/L potassium chloride solution to induce diastolic arrest and then weighed. They were further sectioned and the base of the hearts (including the valves) were immersion-fixed in 4% paraformaldehyde for paraffin embedding and histomorphometry analysis while the apex was snap-frozen in liquid nitrogen and later processed for molecular biology (Western blotting and reverse transcription quantitative polymerase chain reaction of RAS components). Kidneys were collected at P10 and immersion-fixed in 4% paraformaldehyde, cut in half (long-axis sagittal cut), and embedded in paraffin for histomorphometry. More details about the histological and molecular biology methods used in this study are provided in the Supplemental Material.

### Statistical Analysis

We used a total number of 36 litters, 18 per study age (P10 and P28). A sample size of n=6 rats per group was used for all statistical analyses based on previous works from our laboratory using this rat model.^18,20^ Experimental groups were compared by 2-way ANOVA followed by Bonferroni posthoc test, having oxygen exposure (high O_2_ versus room air) and treatments (vehicle cyclodextrin, cyclodextrin–Angio-(1–7), and cyclodextrin-alamandine) as factors. Growth curve was analyzed using 2-way ANOVA for repeated measures. In the 3D computational anatomy study, PCA were used to identify the anatomic models of variation, and differences in shape descriptors (ie, PCA modes) were studied with an unpaired *t* test. The software GraphPad Prism version 5.00 for Windows (GraphPad Software, San Diego, CA) was used for all tests performed. The significance level was established at *P*<0.05.

## Results

### Assessment of Potential Adverse Effects of Neonatal Treatments on Body and Organ Weights and Renal Morphology

Treatments with cyclodextrin–Angio-(1–7) or cyclodextrin-alamandine did not significantly change the weight of the pups in high O_2_-exposed or room air groups throughout time in comparison to vehicle cyclodextrin (Figure S3). We also assessed potential adverse effects of treatments on the renal morphology of neonatal rats. Renal cortex width and glomerular diameter were not significantly changed by treatments in rats kept in room air (Figure S4). The only effect observed was a significant increase in renal cortex width in high O_2_-exposed rats treated with cyclodextrin–Angio-(1–7) versus cyclodextrin-alamandine without reaching statistical significance when compared to vehicle cyclodextrin controls, indicating a possible positive side effect of this drug in exposed rats.

### Ventricular Geometric Changes in Neonatal Rats

**Figure 1.**
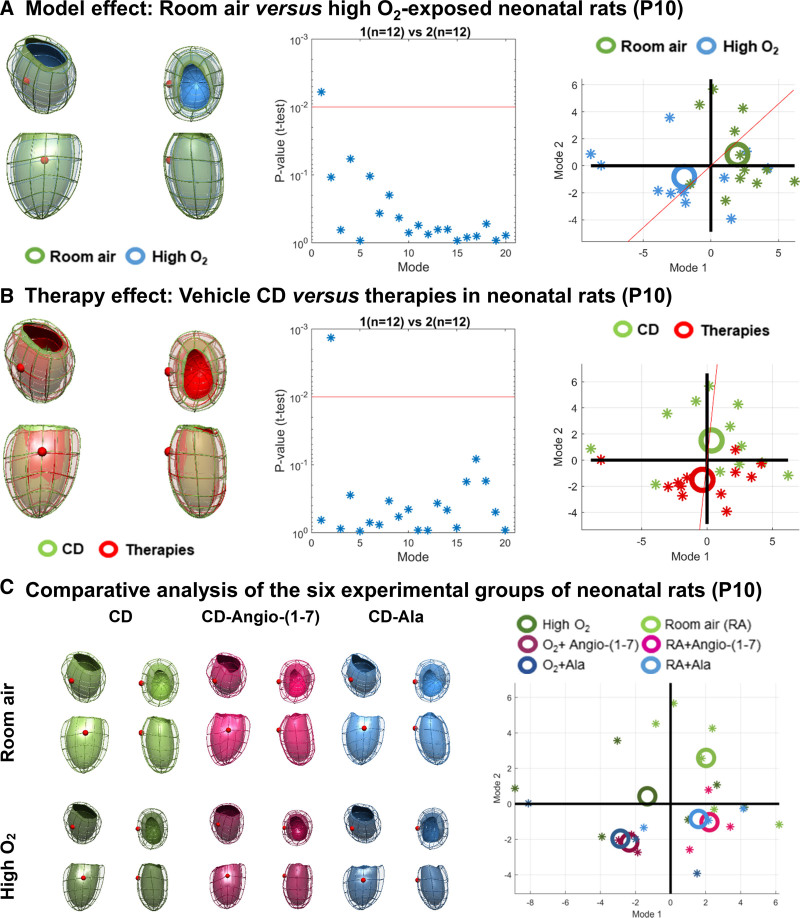
**Computational geometric ventricular analyses of neonatal rats (P10). A**, Analysis of the effect of high-oxygen (O_2_) stress exposure on the anatomy of neonatal rats kept at room air (RA, green) vs high O_2_-exposed (blue) at P10: illustration of the average left ventricular anatomy of each group, statistical comparison of principal component analysis (PCA) modes and a graph indicating individual values (stars) and averages (large circles) per group comparing PCA mode 1 and 2 (n=12 per exposure group). **B**, Analyses of the effect of the treatments, comparing neonatal rats at P10 kept at room air and high O_2_-exposed that were either treated with vehicle cyclodextrin (CD) or with any inclusion compounds (n=12 per exposure group). **C**, Average ventricular anatomy of the six experimental groups and graph with individual values (stars) and averages per group (circles) comparing PCA mode 1 and 2 (n=3 per treatment per exposure group). Red sphere in 3-dimensional meshes points in the direction of the left ventricle lateral wall. Ala indicates alamandine; and Angio, angiotensin.

We first examined the effect of high O_2_ exposure and treatments with cyclodextrin–Angio-(1–7) or cyclodextrin-alamandine on ventricular geometric characteristics (shape and size) of neonatal rats at P10, that is, immediately after high-O_2_ exposure and treatments. High O_2_ exposure was linked to smaller and shorter ventricles with a globular pattern; the characteristics encoded in mode 1 (significant differences between room air and high-O_2_ conditions, see Figure 1A). However, the analyses of all rats (room air and high O_2_-exposed) treated either with vehicle cyclodextrin or any inclusion compounds indicated that treatments triggered significant differences in mode 2 (Figure 1B), leading to smaller and shorter ventricles with a subtle shift of the apex towards the septum (opposite to the free lateral wall).

The average ventricular anatomy of the six experimental groups identified structural alterations compatible with mode 2 induced by cyclodextrin–Angio-(1–7) and cyclodextrin-alamandine treatments in both groups exposed to room air and high O_2_, although they were more pronounced in rats kept at room air than in rats exposed to high O_2_ (Figure 1C). In high O_2_-exposed rats, cyclodextrin–Angio-(1–7) treatment induced a more significant difference in mode 2 compared to cyclodextrin-alamandine and vehicle cyclodextrin, indicating an even smaller but slightly less globular LV shape, with reduced ventricular chamber diameter in high O_2_-exposed rats treated with cyclodextrin–Angio-(1–7).

### Ventricular Functional Changes in Neonatal Rats

**Figure 2.**
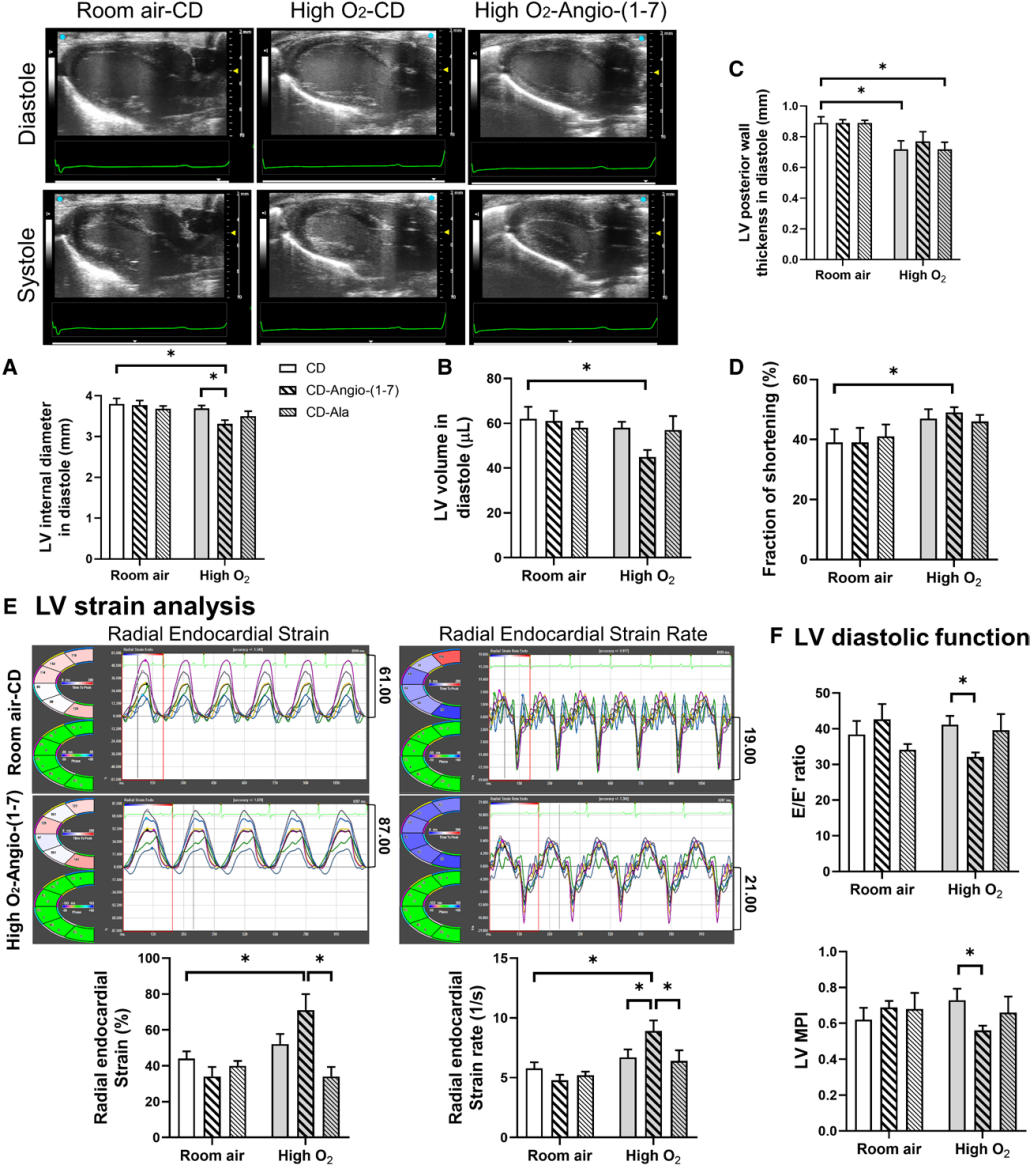
**Echocardiography data of neonatal hearts (P10).** Representative bidimensional long-axis images of the left ventricle (LV) of neonatal rats at P10 kept in room air and treated with vehicle cyclodextrin (CD) vs rats exposed to high O_2_ and treated with CD or Angio/Ang (angiotensin)-(1–7). Echocardiographic parameters measured in M-mode images of all groups of neonatal rats at P10 include (**A**) LV internal diameter in diastole (mm), (**B**) LV volume in diastole (µL), (**C**) LV posterior wall thickness in diastole (mm), and (**D**) fraction of shortening (%). **E**, LV strain analysis with representative image of radial endocardial strain and strain rate in neonatal rats kept in room air and treated with CD vs rats exposed to high O_2_ and treated with Angio-(1–7). Comparisons of radial endocardial strain (%) and strain rate (1/s) between all groups of neonatal rats at P10. **F**, Comparisons of LV diastolic function measured by mitral valve Doppler E wave/ tissue Doppler E′ wave ratio (E/E′) ratio and LV myocardial performance index (MPI) between all groups of neonatal rats. Data presented as mean±SEM. Two-way ANOVA followed by Bonferroni posthoc test. Ala indicates alamandine. *P<0.05.

LV functional changes were assessed in neonatal rats at P10 by echocardiography using bidimensional M-mode short-axis and tissue Doppler images. Neonatal echocardiographic findings are shown in Figure 2 and detailed in Table S1. No significant difference was observed between groups on heart rate, intraventricular septum thickness in diastole (intraventricular septum thickness in diastole), LV blood volume in systole, or LV stroke volume. High O_2_ exposure reduced LV posterior wall thickness in diastole and the LV mass in neonatal rats (Table S1). However, LV mass when indexed to body weight was no longer statistically different between groups.

Cyclodextrin–Angio-(1–7) and cyclodextrin-alamandine treatments did not induce significant changes in LV function in neonatal rats kept in room air. However, in high O_2_-exposed rats, cyclodextrin–Angio-(1–7) treatment triggered more pronounced positive LV functional changes in comparison to cyclodextrin-alamandine. High O_2_-exposed rats treated with cyclodextrin–Angio-(1–7) had reduced LV internal chamber diameters (LV internal diameter in diastole, Figure 2A) and blood volume in diastole (Figure 2B), which are features compatible with the smaller and less globular shape identified in the 3D geometric analyses. Furthermore, cyclodextrin–Angio-(1–7) prevented reduced LV posterior wall thickness in diastole in high O_2_-exposed rats (Figure 2C), although it significantly reduced LV mass in these rats in comparison to rats kept in room air treated with vehicle cyclodextrin (Table S1). However, LV mass when indexed to body weight was no longer different between groups.

Cyclodextrin–Angio-(1–7) treatment markedly improved LV systolic function in neonatal high O_2_-exposed rats. This improvement was indicated by an average 10% higher fraction of shortening (Figure 2D) and a significant increment of radial endocardial strain in this group in comparison to neonatal rats kept in room air treated with vehicle cyclodextrin (Figure 2E). Similarly, the radial endocardial strain rate was also markedly increased in high O_2_-exposed rats treated with cyclodextrin–Angio-(1–7) versus room air and high O_2_–exposed rats treated with vehicle cyclodextrin (Figure 2E), supporting enhanced myocardial contractility in cyclodextrin–Angio-(1–7) treated group. We also observed improvements in LV diastolic function (Figure 2F) in high O_2_-exposed rats treated with cyclodextrin–Angio-(1–7). The significant reduction in LV mitral valve Doppler E wave/ tissue Doppler E′ wave ratio (E/E′) ratio in this group indicates a better balance between ventricular relaxation and filling pressures (Figure 2F). This is corroborated by a significantly lower LV myocardial performance index (MPI) in this group (Figure 2F), indicating enhanced global systolic and diastolic functions in high O_2_-exposed rats treated with cyclodextrin–Angio-(1–7) versus vehicle cyclodextrin.

Cyclodextrin-alamandine treatment did not induce any significant LV functional changes in neonatal rats with features very similar to rats treated with vehicle cyclodextrin in both exposure groups (Table S1).

### Histomorphological Ventricular Changes in Neonatal Rats

**Figure 3.**
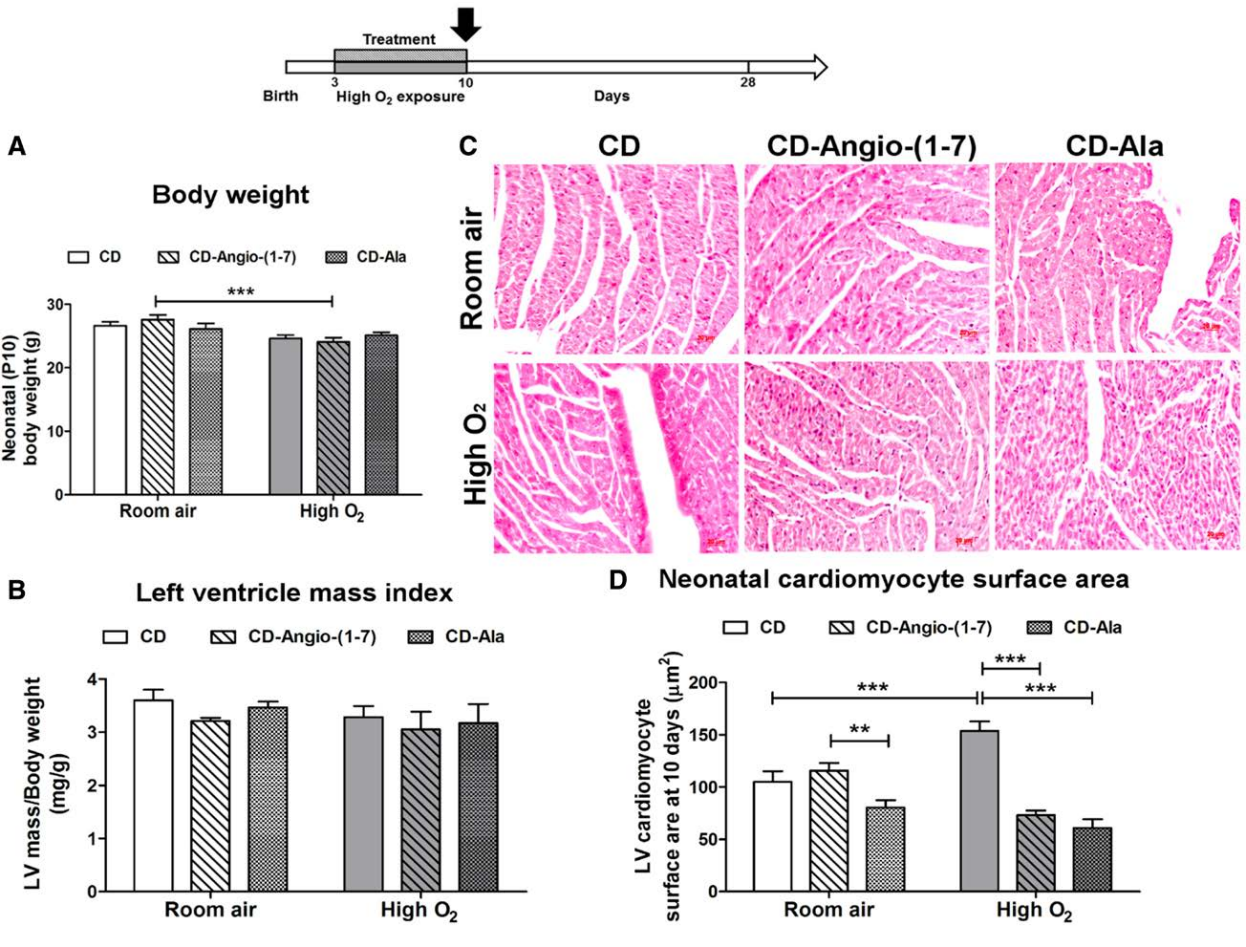
**Histomorphometry analyses of neonatal hearts (P10).** Study design showing hearts collected at day 10 of life (P10) after 7 d of oxygen exposure and treatment interventions in neonatal rats. **A**, Bodyweight and (**B**) left ventricular (LV) mass per body weight index of neonatal rats at P10. **C**, Representative histological images and (**D**) quantification of LV cardiomyocytes surface area of the hearts of neonatal rats (n=6/group) exposed to high-O_2_ or kept at room air and treated with vehicle cyclodextrin (CD) or included CD-Angio (angiotensin)-(1–7) or CD-alamandine (Ala). Data presented as mean±SEM. Two-way ANOVA followed by Bonferroni posthoc test. *P<0.05, **P<0.01 and ***P<0.001.

At P10, high O_2_ exposure and drug treatments did not induce significant changes in body weight in comparison to rats kept at room air and treated with vehicle cyclodextrin (Figure 3A). The only statistically significant difference observed between groups was a lower weight in pups exposed to high O_2_ treated with cyclodextrin–Angio-(1–7) versus pups kept in room air receiving the same treatment. Cardiac index, calculated by the ratio of the LV weight (extracted postmortem) to body weight, was not significantly different between groups (Figure 3B).

Histological analyses of P10 hearts showed increased average LV cardiomyocyte surface area as an indication of hypertrophy in high O_2_-exposed rats versus room air rats treated with vehicle cyclodextrin (Figure 3C and 3D). However, this effect was removed by treating high O_2_-exposed rats with cyclodextrin–Angio-(1–7) or cyclodextrin-alamandine. In rats kept at room air, both cyclodextrin–Angio-(1–7) treatment and cyclodextrin-alamandine did not significantly change cardiomyocyte surface area in comparison to rats treated with vehicle cyclodextrin.

### Long-Term Effects of Neonatal Treatments on Ventricular Function and Histomorphologic Changes

**Figure 4.**
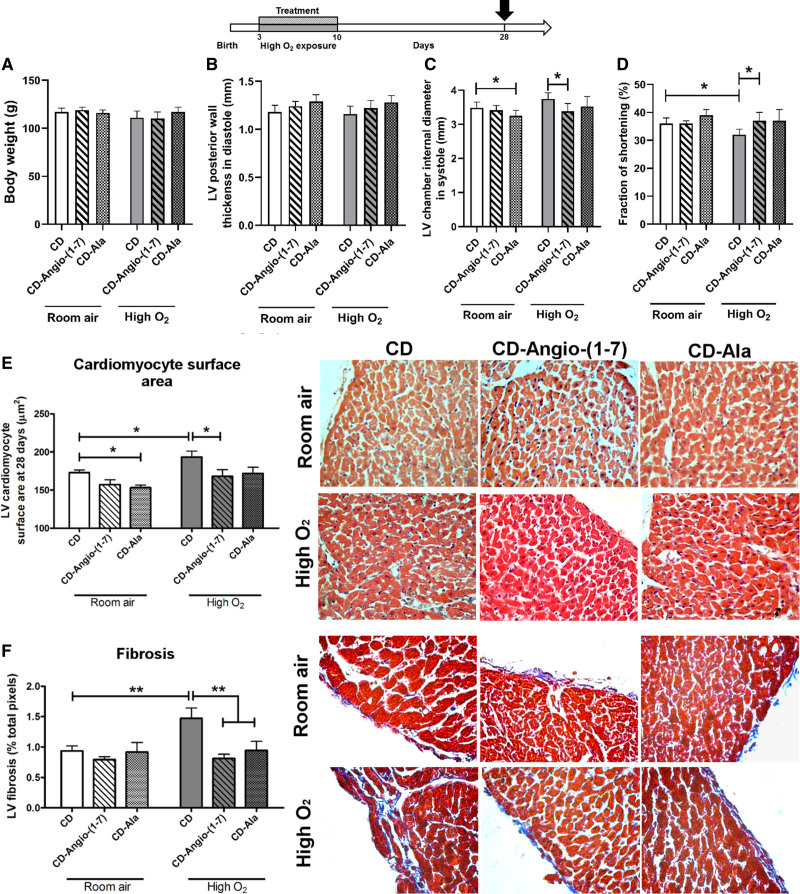
**Echocardiography and histomorphometry analyses of juvenile hearts (P28).** Study design showing hearts collected at day 28 of life (P28) following previous exposure to high-O_2_ or room air and treatment with vehicle cyclodextrin (CD) or included CD-Angio (angiotensin)-(1–7) or CD-alamandine (Ala; n=6/group). **A**, Bodyweight at P28. Echocardiographic parameters measured in bidimensional M-mode images of rats at P28 include (**B**) left ventricular (LV) posterior wall thickness in diastole, (**C**) LV chamber internal diameter in systole, and (**D**) fraction of shortening. Histology measurements and representative images include (**E**) LV cardiomyocyte surface area and (**F**) LV myocardium interstitial fibrosis. Data presented as mean±SD. Two-way ANOVA followed by Bonferroni posthoc test. *P<0.05 and **P<0.01.

We examined whether neonatal treatment with cyclodextrin–Angio-(1–7) and cyclodextrin-alamandine could induce long-term positive effects on LV function and remodeling assessed in juvenile rats at P28. LV functional changes were assessed in juvenile rats at P28 by echocardiography using a similar protocol as for P10 with bidimensional M-mode short-axis and tissue Doppler images to obtain wall thickness and chamber diameter measurements and to calculate the fraction of shortening (Table S2 and Figure 4). We have previously shown that high O_2_-exposed rats can develop impaired systolic function at this age, mainly characterized by reduced fraction of shortening with increased myocardium hypertrophy and fibrosis.^18,20^ No significant difference was observed for body weight (Figure 4A) or LV posterior wall thickness in diastole (Figure 4B) between groups. Furthermore, no significant difference was observed between juvenile rats previously exposed to room air and high O_2_ on left ventricle internal diameter in systole (Figure 4C). Cyclodextrin-alamandine treatment, however, significantly reduced this parameter in room air rats and cyclodextrin–Angio-(1–7) in high O_2_-exposed rats in comparison to rats previously kept at room air treated with vehicle cyclodextrin. Fraction of shortening was significantly reduced in juvenile rats previously exposed to high O_2_ versus room air, which was prevented by neonatal treatments with cyclodextrin–Angio-(1–7) and cyclodextrin-alamandine treatments (Figure 4D).

LV cardiomyocyte surface area was significantly higher in juvenile rats previously exposed to high O_2_ versus room air, indicative of cardiomyocyte hypertrophy (Figure 4E). However, this effect was completely prevented by neonatal treatment with cyclodextrin–Angio-(1–7). In juvenile rats previously kept at room air, only alamandine treatment significantly reduced cardiomyocyte surface area. Both neonatal treatments had also markedly reduced LV fibrosis in juvenile rats previously exposed to high O_2_ without significant effects in rats kept in room air (Figure 4F).

### Cardiac RAS Changes in Neonatal Rats

**Figure 5.**
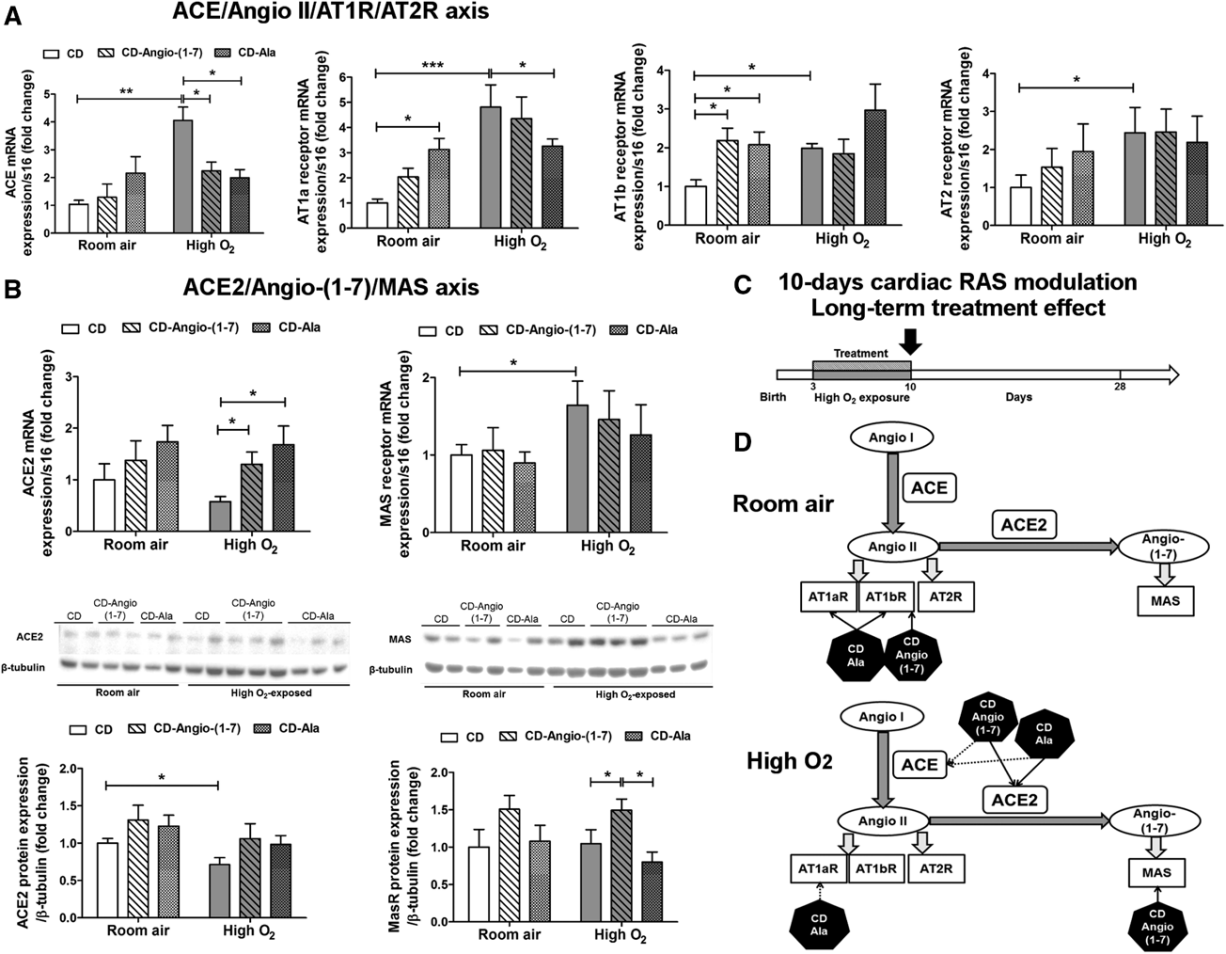
**Cardiac renin-angiotensin system (RAS) changes in neonatal rats (P10). A**, mRNA expression of components of the ACE (angiotensin-converting enzyme)/Angio II (angiotensin II)/AT1a (angiotensin receptor type 1a), AT1b and AT2 (angiotensin receptor type 2) receptors axis, and (**B**) mRNA and protein expressions of ACE2 and Mas receptor in left ventricle (LV) of neonatal rats at P10 (n=6/group) exposed to high O_2_ or at room air and treated with vehicle cyclodextrin (CD) or included CD-Angio-(1–7) or CD-alamandine (Ala). Data presented as mean±SEM. Two-way ANOVA followed by Bonferroni posthoc test. *P<0.05, **P<0.01 and ***P<0.001. **C**, Study design indicating that hearts were collected at P10. **D**, Summary of cardiac RAS findings indicating which treatment (black hexagons) has stimulated (arrow with full line) or inhibited (arrow with dotted line) RAS components in neonatal rats kept at room air or high O_2_. AT1R indicates angiotensin II receptor type 1; AT2R, angiotensin II receptor type 2; AT1aR, angiotensin II type 1 receptor a; AT1bR, angiotensin II type 1 receptor b; MAS, Mas receptor protein; and MasR, Mas receptor gene.

We assessed changes in cardiac RAS profile at P10 in neonatal rats treated with cyclodextrin–Angio-(1–7) or cyclodextrin-alamandine (Figure 5). Figures 5A describes differences in *ACE*, *AT1a*, *AT1b*, and *AT2* mRNA expression in ventricles. *ACE* mRNA expression was significantly higher in high O_2_-exposed rats treated with vehicle cyclodextrin at P10, which was prevented by both treatments (Figure 5A). *AT1a* mRNA expression was similarly higher in high O_2_-exposed neonatal rats treated with vehicle cyclodextrin (Figure 5A). This was prevented only by cyclodextrin-alamandine treatment. However, in rats kept in room air, cyclodextrin-alamandine treatment increased cardiac *AT1a* mRNA expression (Figure 5A). This paradoxical response of cyclodextrin-alamandine between rats kept at room air and high O_2_ suggests that this peptide may indirectly regulate the transcription of AT1a gene in ventricles. High O_2_ exposure also increased the mRNA expression of *AT1b* (Figure 5A) and *AT2* (Figure 5A), and no treatment significantly affected their transcription in high O_2_-exposed rats. However, cyclodextrin–Angio-(1–7) and cyclodextrin-alamandine treatments increased *AT1b* mRNA expression in ventricles of neonatal rats kept in room air (Figure 5A), also suggesting an indirect effect of these peptides on *AT1b* gene regulation.

Figures 5B shows results of ACE2 and Mas receptor protein and mRNA expressions. High O_2_ exposure did not change cardiac *ACE2* mRNA expression in neonatal rats treated with vehicle cyclodextrin but significantly reduced ACE2 protein expression (Figure 5B). Cyclodextrin–Angio-(1–7) and cyclodextrin-alamandine treatments markedly increased *ACE2* mRNA expression but not the protein expression in ventricles of neonatal rats exposed to high O_2_ (Figures 5B). The mRNA expression of Mas receptor was significantly increased in neonatal rats exposed to high O_2_ compared to room air, while its protein expression was not different (Figure 5B). Only cyclodextrin–Angio-(1–7) treatment significantly increased the protein expression of Mas receptor in high O_2_-exposed rats (Figure 5B). A summary of our findings in ventricles of neonatal rats is shown in Figure 5C and 5D. Briefly, they indicate an inverse regulation of ACE and ACE2, prevailing the upregulation of ACE2, by cyclodextrin–Angio-(1–7) and cyclodextrin-alamandine treatments in neonatal rats immediately after high O_2_ exposure without significant downregulation of AT1 receptor genes.

### Long-Term Changes in Cardiac RAS Induced by Neonatal Treatments

**Figure 6.**
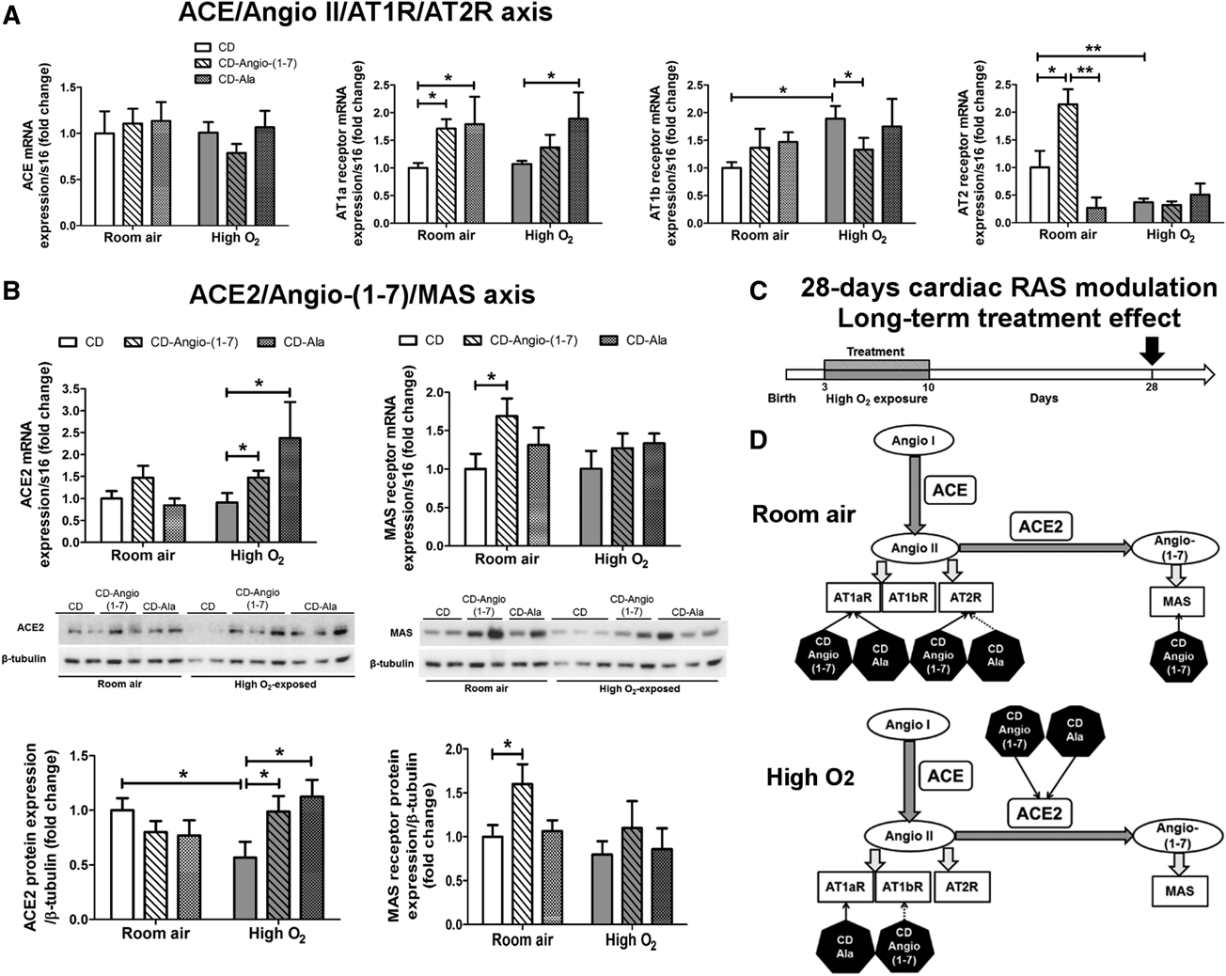
**Cardiac renin-angiotensin system (RAS) changes in juvenile rats (P28). A**, mRNA expression of components of the ACE (angiotensin-converting enzyme)/Angio II (angiotensin II)/AT1a (angiotensin receptor type 1a), AT1b (angiotensin receptor type 1b), and AT2 (angiotensin receptor type 2) receptors axis, and (**B**) mRNA and protein expressions of ACE2 and Mas receptor in left ventricle (LV) of juvenile rats at P28 (n=6/group) exposed to high O_2_ or at room air and treated with vehicle cyclodextrin (CD) or included CD-Angio-(1–7) or CD-alamandine (Ala) from P3 to P10. Data presented as mean±SEM. Two-way ANOVA followed by Bonferroni posthoc test. *P<0.05 and **P<0.01. **C**, Study design indicating that hearts were collected at P28. **D**, Summary of cardiac RAS findings indicating which treatment (black hexagons) has stimulated (arrow with full line) or inhibited (arrow with dotted line) RAS components in juvenile rats kept at room air or high O_2_ from P3 to P10. AT1R indicates angiotensin II receptor type 1; AT2R, angiotensin II receptor type 2; AT1aR, angiotensin II type 1 receptor a; AT1bR, angiotensin II type 1 receptor b; and MAS, Mas receptor protein.

We examined the same RAS components in the LV of juvenile rats at P28, previously exposed to high O_2_ or kept at room air and treated with cyclodextrin–Angio-(1–7) or cyclodextrin-alamandine from P3 to P10 (Figure 6). No difference was observed in *ACE* mRNA expression between groups (Figure 6A). Cyclodextrin–Angio-(1–7) and cyclodextrin-alamandine treatments significantly increased *AT1a* mRNA expression compared to vehicle cyclodextrin in rats kept at room air, whereas only alamandine treatment increased it in the LV of rats previously exposed to high O_2_ (Figure 6A). *AT1b* mRNA expression was increased in LV of juvenile rats exposed to high O_2_ but prevented only by cyclodextrin–Angio-(1–7) treatment (Figure 6A). *AT2* mRNA expression was significantly lower in LV of juvenile rats previously exposed to high O_2_ and both treatments did not change this low transcription in juvenile rats (Figure 6A). However, in rats previously kept at room air, only early cyclodextrin–Angio-(1–7) treatment significantly increased long-term cardiac *AT2* mRNA expression in comparison to rats treated with vehicle cyclodextrin (Figure 6A).

Juvenile rats previously exposed to high O_2_ did not have *ACE2* mRNA expression changes in LV but had lower protein expression of ACE2 (Figure 6B). Both cyclodextrin–Angio-(1–7) and cyclodextrin-alamandine treatments significantly increased *ACE2* mRNA and protein expressions in LV in juvenile rats previously exposed to high O_2_. No significant change was observed in Mas receptor mRNA and protein expressions in ventricles of juvenile rats previously exposed to high O_2_ (Figure 6B). However, cyclodextrin–Angio-(1–7) treatment markedly increased long-term mRNA and protein expressions of Mas receptor in ventricles of juvenile rats kept at room air but not in high O_2_-exposed rats, whereas cyclodextrin-alamandine had no significant effect (Figures 6B). A summary of the main findings in ventricles of juvenile rats at P28 is shown in Figure 6C and 6D. Briefly, they indicate a long-term upregulation of ACE2 in ventricles of juvenile rats previously exposed to high O_2_ and treated with either cyclodextrin–Angio-(1–7) or cyclodextrin-alamandine.

## Discussion

In this study, we showed that treatment with cyclodextrin–Angio-(1–7) and cyclodextrin-alamandine were effective at preventing pathological cardiac remodeling in juvenile rats exposed to high-O_2_ during the neonatal period. Of the 2 treatments, cyclodextrin–Angio-(1–7) presented the most significant positive effects on LV remodeling and function in neonatal rats exposed to high O_2_. In addition, although cyclodextrin–Angio-(1–7) induced significant changes in ventricular geometry, remodeling, and function, cyclodextrin-alamandine effectiveness was limited to the reduction of LV myocardium hypertrophy in neonatal rats and in overall remodeling in juvenile rats exposed to high O_2_. Importantly, no treatment significantly downregulated or blocked Angio II AT1 receptor expression, whereas both cyclodextrin–Angio-(1–7) and cyclodextrin-alamandine reduced *ACE* gene expression to values similar to controls. In high-O_2_ animals, ACE2 was similarly stimulated by both treatments through gene upregulation in neonatal and juvenile rats.

The 3D computational cardiac images revealed altered ventricular shape and remodeling at neonatal age (P10) in rats exposed to high-O_2_, mainly characterized by small and short ventricles with a more globular pattern. Histomorphometry further indicated that this pattern was associated with cardiomyocyte hypertrophy in high O_2_-exposed rats, which may represent a myocardial compensatory adaptation to maintain the cardiac output in such small hearts. Still, LV function was preserved at neonatal age in exposed rats. These changes in ventricular shape and remodeling were also shown in our study to predispose the developmental programming of cardiomyopathy in O_2_-exposed rats when reaching juvenile age. Interestingly, both treatments failed to rescue the smaller and shorter pattern of ventricles in O_2_-exposed rats; however, cyclodextrin–Angio-(1–7) has more effectively reshaped it to a slightly smaller aspect with a consequent reduction of ventricular diastolic volume. Compensatory improvements for systolic and diastolic ventricular function and myocardium contractility were observed in neonatal rats exposed to high O_2_ treated with vehicle cyclodextrin and the inclusion compounds, probably due to their lower ventricular volumes. However, LV functional improvements were more remarkable and not associated with cardiomyocyte hypertrophy only in neonatal rats treated with cyclodextrin–Angio-(1–7). Cyclodextrin-alamandine, however, did not induce changes in ventricular shape and function as observed with cyclodextrin–Angio-(1–7) treatment, maintaining instead a pattern and functional parameters that were very close to O_2_-exposed rats treated with vehicle. However, cyclodextrin-alamandine still effectively reduced cardiomyocyte surface area and hypertrophy in high O_2_-exposed neonatal rats. Although we observed subtle geometric changes in control groups treated with the inclusion compounds, they have not associated with significant LV remodeling and dysfunction.

Our ventricular geometric results are similar to clinical findings in preterm-born infants. Previously, a large study performing echocardiographic imaging in >390 preterm and full-term born newborns described smaller hearts in preterm newborns with disproportionate catch-up growth in the first 3 months of life.^8^ In this study, a similar method of computational ventricular analysis was performed from bidimensional ultrasound imaging, revealing in preterm babies a ventricular pattern consistent with a globular shape. In line with these findings, Cox et al^5^ have used cardiac magnetic resonance scans of preterm-born neonates and computational atlases to also demonstrate a ventricular globular shape with spherical blood pool in preterm-born neonates. Similar to our findings, early in life cardiac remodeling was described to persist into adulthood in preterm-born individuals. This was first reported by Lewandowski et al,^28^ where they showed short-patterned LV associated with dysfunction and higher LV mass in young adults born prematurely compared to those born full-term. LV dysfunction was further linked to greater diffuse myocardial fibrosis in the LV of young adults born preterm, which relates to the degree of prematurity.^29^

Hence, our rat model successfully reproduced the main cardiac features observed in preterm-born young adults that may be predisposing them to an accelerated progression towards dysfunction and disease.

Crispi et al^30^ previously described the impact of intrauterine ventricular remodeling on perinatal and postnatal growth outcomes in small for gestational age newborns.^31^ Their findings also revealed that globular and hypertrophied ventricular patterns in fetal hearts can associate with sustained hemodynamic and ventricular diastolic dysfunction into postnatal life^32^ and up to preadolescence.^33^ However, another fetal ventricular pattern described by them that is consistent with an elongated shape has been associated with better outcomes.^31^ These findings indicate that early compensatory ventricular patterns could lead to better outcomes in this population.

In our rat model, all O_2_-exposed groups developed shorter ventricles in comparison to room air controls, indicating an activation of O_2_-sensitive mechanisms capable of regulating cardiac development that was not rescued by any treatment. However, our findings indicate that the main positive responses triggered by both treatments in O_2_-exposed rats were related to the inhibition of cardiomyocyte hypertrophy and ventricular fibrosis rather than rescuing the smaller and shorter LV shape observed in high O_2_-exposed rats. Therefore, our treatments targeting the cardioprotective RAS axis revealed an important effect of this system on heart plasticity during neonatal life.

The RAS is a well-recognized central mechanism regulating blood pressure through the actions of vasoactive peptides that can also control blood volume. In fetal life, RAS components such as Angio II and AT1 receptors are essential for kidney and heart formation and maturation, notably in the last gestational trimester.^24,34–36^ However, in prematurity, this system can be overactivated, which has been shown to trigger a paradoxical negative effect by stimulating an accelerated organ maturation and remodeling.^21^ Our results showed that restoring the balance of this system during development can be beneficial to prevent heart diseases later in life in our animal model. More importantly, we have not observed any adverse effects of Angio-(1–7) or alamandine treatments in neonatal rats. We have only reported a significant increase in renal cortex width in neonatal rats exposed to high O_2_ treated with Angio-(1–7), which is a positive effect. A previous study from our laboratory has shown that hyperoxia exposure resulted in a significant reduction in both nephrogenic zone width and glomerular diameter at P5 in this model, and a significantly increased apoptotic cell count, without affecting nephron number at P10. We also observed that the expression of the HIF (hypoxic-inducible factor)-1α was significantly reduced in the developing kidney following hyperoxia exposure.^37^ In this study, a systemic administration of the HIF-1α stabilizer dimethyloxalylglycine resulted in enhanced expression of HIF-1α and improved nephrogenesis in hyperoxia-exposed pups mainly by increasing nephrogenic zone width and glomerular diameter. However, interactions between the RAS and particularly of Angio-(1–7), and HIF-1α are still unclear, especially in the kidney.

In our study, both treatments were effective at preventing programming of cardiomyopathy in juvenile rats previously exposed to high O_2_. Over the years, the cardioprotective effect of Angio-(1–7) has emerged as a promising therapeutic avenue for the management of several pathological outcomes.^22^ Angio-(1–7)–mediated antifibrogenic and antihypertensive effects have been systematically reported in different experimental models of hypertension and heart diseases and also when using a similar orally active formula to deliver this peptide.^25,38,39^ In addition, the recently discovered alamandine peptide is shown to stimulate cardioprotective responses comparable to those of Angio-(1–7) through mechanisms that also involve the reduction of cardiac fibrosis and blood pressure in a heart failure model.^22^ This was further confirmed by Oliveira et al^40^ describing a severe programming of dilated cardiomyopathy in a mice deficient in alamandine’s receptor MrgD (Mas-related G protein-coupled receptor member D).

A common positive effect of both treatments observed in our study was the upregulation of cardiac ACE2 in O_2_-exposed rats. Although our rats were only treated during the neonatal period, this effect was shown as long-lasting and associated with better ventricular remodeling and function in rats into adulthood. ACE2 is a well-known mechanism shifting the RAS deleterious actions towards its cardioprotective axis, prevailing Angio-(1–7) actions rather than Angio II.^41,42^ ACE2 has also been considered a potential target molecule for therapies because of its extensive distribution within the cardiovascular system. By cleaving Angio II and forming Angio-(1–7), ACE2 further bypasses the activation of AT1 receptors.^41,42^ This was previously shown by others when treating ACE2 knockout mice infused with Angio II with an exogenous human recombinant ACE2.^43^ It stimulated a dramatic decrease on plasmatic and myocardial Angio II levels while increasing Angio-(1–7) in treated mice. In our study, both treatments have sustained a balance in favor of ACE2. Acutely, they have mainly stimulated ACE2 gene expression, whereas in the long-term, both therapies caused a sustained gene and protein ACE2 overexpression in O_2_-exposed rats, indicating potential epigenetic modifications targeting this gene induced by early treatment with both peptides. However, the molecular or genetic mechanisms up-regulating ACE2 triggered by these treatments are still unclear.

Novel downstream players involved in the biological differences and similarities between Angio-(1–7) and alamandine are not fully understood. This is a fast-advancing field of research that faces additional challenges in newborns when RAS plays an essential but also dueling role under stress. The positive effects of cyclodextrin–Angio-(1–7) and cyclodextrin-alamandine treatments observed in our animal model of neonatal O_2_-stress represent an important and novel therapeutic avenue to prevent the programming of diseases in prematurity.

### Strengths and Limitations

This is the first study describing the direct impact of environmental stress such as high-O_2_ exposure and treatments on neonatal heart remodeling, at a time when heart development is still ongoing in rats. The description of different ventricular shape patterns was possible due to the creation of a 3D computational method using high-frequency ultrasound imaging of neonatal rats at P10. The 3D model was further complemented by detailed ventricular histomorphometry and functional assessments. Previous limitations to assess neonatal ventricular shape and function in rodents were probably due to their small ventricle size (≈8 mm) and accelerated heart rate, which can quickly and significantly vary in response to environmental factors such as anesthesia and temperature changes, for example. Therefore, image acquisition requires a fast recording and experienced personnel. When using a high-frequency ultrasound probe in neonatal rats, these problems are compensated by anatomic characteristics such as thin skin and lower thoracic bone density, which significantly facilitated the 3D screening of neonatal ventricles. These anatomic characteristics were also crucial in our study to warranty the quality of our images and contours during the segmentation process. Still, the quality of some images obtained from the ventricular base (area close to the valves) were compromised by the sternum superposition. This issue also made recording of 3D ventricular images in juvenile rats in our study unfeasible, mainly due to their higher bone density in the thoracic cage. Nevertheless, our main goal in this study was to record neonatal images and to associate ventricular patterns with later-in-life adaptive or nonadaptive cardiac function into adulthood. Therefore, in juvenile rats, we performed the same echocardiographic method using the same equipment as in our previous studies, in which we described for the first time the programming of cardiomyopathy induced by neonatal exposure to high O_2_.^18,20^

Another important strength of our study was the use of an orally active formulation of cyclodextrin–Angio-(1–7) and cyclodextrin-alamandine included in β-cyclodextrins to treat neonatal rats. The structure of the inclusion compound protects the internal peptide against proteolytic enzymes present in the stomach and intestine.^44^ In addition, cyclodextrins are enzymatically degraded by bacterial enzymes at the distal portions of the digestive tract, releasing the peptide that is mainly absorbed in the colon. This therapeutic strategy could therefore more closely mimic the clinical scenario for nasogastric tube feeding and treatment of preterm newborns.

An important limitation of this study was the use of only male rats in our analyses. This was justified by the fact that female rats exposed to neonatal high O_2_ stress do not present significant cardiac remodeling and functional changes before the increment of blood pressure (data provided in Supplemental Material), which happens only after 7 weeks of age in this model.^19^ Blood pressure changes may, therefore, be an important factor triggering decompensation with heart changes and dysfunction in female rats in this model. Further studies from our group are needed to elucidate sex-based differences in the developmental programming of cardiac diseases using this model.

### Perspectives

Our findings have used a newly developed method for 3D geometric ventricular analyses to investigate ventricular shape differences and cardiac remodeling induced by neonatal high O_2_ stress and treatments targeting the cardioprotective RAS axis in rats. The observed different ventricular remodeling patterns may indicate a higher predisposition of hyperoxia-exposed neonatal rats to develop cardiomyopathy into adulthood, which could be modified or prevented by cyclodextrin-alamandine and more significantly by cyclodextrin–Angio-(1–7) treatments. In both treatments, the upregulation of cardiac ACE2 could also be key to balancing RAS and preventing the deleterious effects of Angio II/AT1 on heart remodeling and programming of cardiomyopathy in this model of neonatal stress.

Taken together, our findings describe an important cardiac plasticity in neonatal rats in response to stressful conditions and therapies. Furthermore, we have also shown that the neonatal period may be an important intervention window to effectively prevent ventricular remodeling and the programming of cardiomyopathy in preterm-born subjects.

## Article Information

### Acknowledgments

We thank the scientific team of the Institutos Nacionais de Ciência e Tecnologia-Nanobiofar in Brazil for providing the inclusion compounds and peptides used in our experiments. We also thank the staff team of the Centre Hospitalier Universitaire Sainte-Justine animal facility for their support in our experiments and imaging recordings.

### Sources of Funding

Heart and Stroke Foundation of Canada (Quebec) Grants-in-aid (A.M. Nuyt), Canadian Institutes of Health Research (CIHR) grants MOP220771 (A.M. Nuyt) and Canada Fund for Innovation (A.M. Nuyt). R.A.S. Santos was supported by a Conselho Nacional de Desenvolvimento Científico e Tecnológico-Institutos Nacionais de Ciência e Tecnologia. grant and FAPEMIG–Fundação de Amparo à Pesquisa do Estado de Minas Gerais (APQ-03139-16), D.R. Dartora was supported by a scholarship from the Coordination for the Improvement of Higher Education Personnel (CAPES); Ministry of Education in Brazil (Process number: 99999.011682/2013-02). M. Bertagnolli was supported by the Bourse d’excellence pour étudiants étrangers du Fonds québécois de la recherche en santé (FRQ-S), a Jacques-de Champlain/Société Québécoise d’Hypertension Artérielle fellowship award and a SickKids Foundation grant (NI20-1037). A.J. Lewandowski was supported by a British Heart Foundation Intermediate Basic Science Research Fellowship (FS/18/3/33292). P. Lamata was funded in part by the Wellcome Trust (209450/Z/17/Z). For the purpose of Open Access, the author has applied a CC BY public copyright license to any author accepted article version arising from this submission.

### Disclosures

None.

## Supplementary Material


