# Supplementary material for: Reshaping the Preterm Heart: Shifting Cardiac Renin-Angiotensin System Towards Cardioprotection in Rats Exposed to Neonatal High-Oxygen Stress
Source: Hypertension. 2022 May 19;79(8):1789–803. doi: 10.1161/HYPERTENSIONAHA.122.19115 (PMC9278707; doi:10.1161/HYPERTENSIONAHA.122.19115)
Supplement: Supplementary file 1 [file hyp-79-1789-s001.pdf]

SUPPLEMENTAL MATERIAL

Reshaping the preterm heart: Shifting cardiac renin-angiotensin system towards cardioprotection in rats exposed to neonatal high-oxygen stress

Mariane Bertagnolli, Daniela R. Dartora, Pablo Lamata, Ernesto Zacur, Thuy-An Mai Vo, Ying He, Léonie Beauchamp, Adam J. Lewandowski, Anik Cloutier, Megan Sutherland, Robson A. S. Santos, Anne Monique Nuyt

DETAILED METHODS

Echocardiography in adult rats

At P28, LV structure and function were assessed by echocardiography under isoflurane anesthesia (2:1 O_2) using an ACUSON CV70 ultrasound imaging system (Siemens Medical Solutions, Burlington, ON) equipped with a 12 MHz scan head, as performed and described previously.[1, 2] Briefly, two-dimensional guided M-mode images were obtained from a short-axis LV view at the papillary muscle level. LV internal diameter and wall thickness were measured to calculate the fraction of shortening (FS= 100 x [(LVIDd-LVIDs)/LVIDd]) using a similar method described for P10 image analyses.[2]

Histology

Transverse cross-sections of LV and right ventricles (RV) were paraffin-embedded and 5 µm sections stained with hematoxylin and eosin for measurements of cardiomyocyte surface area. Additional ventricular sections were stained with Masson's Trichrome for cardiac fibrosis quantification. Three pictures were obtained randomly from subendocardium and sub-epicardium areas of the LV. Cardiomyocyte size was evaluated by measuring the perimeter and surface area of cells with a visible nucleus.[1] Cardiac fibrosis was assessed by quantifying the interstitial blue staining pixels (corrected as % of total pixels) obtained from the Masson's trichrome staining. The software Image J 1.36b (http://rsbweb.nih.gov/ij/) was used for stereological analysis and pixels quantification, as described previously.

Five µm sections from the central region of the kidney (across the full coronal plane) were stained with hematoxylin and eosin. To assess renal cortex width, four images of the cortex and medulla were taken with a 10X objective for each section. Using image analysis software (Image J), the width of the renal cortex (from the cortico-medullary junction to the superficial edge of the outer renal cortex) was measured twice in each of the four images, and the average calculated for each kidney. To determine renal glomerulus size, a complete section from each kidney was systematically sampled (with a 40X lens) at a step length of 300 µm. At each field of view, Image J software was used to trace the perimeter of the Bowman's capsule of every glomerulus (> 50 glomeruli were sampled for each kidney).[1] The average cross-sectional area of the renal glomeruli was then calculated for each kidney.

Western Blotting

Hearts were homogenized in RIPA (radioimmuno-precipitation assay buffer) buffer containing protease inhibitors (Na-deoxycholate 10%, EDTA 100 mM, SDS 10%, Complete

mini (Roche) 0.05X, phenylmethanesulfonylfluoride (PMSF) 100mM, Igepal 10%). Antibodies against ACE2 (ab108252, 1/1,000 dilution, Abcam, Cambridge, MA), Mas receptor (AAR-013, 1/1,000 dilution, Alomone, Jerusalem, Israel) were used in this study. Antibody against β-tubulin (T0198, 1:2,500 dilution, Sigma-Aldrich Canada Co., Oakville, ON) was used as control. Protein bands were developed with an enhanced chemiluminescence substrate (PerkinElmer Inc, Waltham, MA) and quantified using Image J.

Reverse transcription - quantitative PCR

The mRNA expression levels of angiotensin receptors were determined by reverse transcription (RT) of total RNA followed by quantitative PCR (qPCR). Total RNA was extracted from LV using RNeasy Mini Kit (Qiagen Inc, Toronto, ON). One µg of total RNA was reversed transcribed using Omniscript RT Kit (Qiagen Inc, Toronto, ON) and qPCR was performed using SYBER Green PCR Master Mix (Applied Biosystems, Carlsbad, CA) with a MX3000P Stratagene real-time PCR cycler (Agilent Technologies, Mississauga, ON, Canada). The following PCR conditions were used: DNA was denatured for 10 min at 95°C followed by 45 cycles of denaturation at 95°C for 15 s and annealing/elongation at 60°C for 1 min. The following cDNAs were amplified with the primers indicated in parentheses. Agtr1a (AT1a) (forward 5'-CCAAGTCCCACTCAAGCCT-3'and reverse 5'-TTGCCAGTGTGCTTTGAACC-3'), Agtr1b (AT1b) (forward 5'-GCACTCTTTCCTACCGCCCT-3' and reverse 5'-CACTTTCTCTGCTTCAACCCTG-3'), Agtr2 (AT2) (forward 5' TGTGTTGGCATTCATCATTTG-3' and reverse 5'-AGAACTGCTTTTTCGGCAAG-3'), ACE (forward 5'-AGATCCTGCTTCAGAAAAACAAG-3'and reverse 5'-TGTCAGATCAGGCTCCAGTG-3') and ACE2 (forward 5'- CACTGACTGGAGCCCATATG-3' and reverse 5'-TCCTGATGGCCTCTTCAACT-3'). The 40S ribosomal protein S16 (Rps16) was used as internal control (forward 5'-TCTGGGCAAGGAGAGATTTG-3'and reverse 5'-CCGCCAAACTTCTTGGATTC-3'). Primers were designed to have a melting temperature (Tm) of 60°C and a 3' GC clamp using Primer3.

SUPPLEMENTAL REFERENCES

1. Bertagnolli M, Dios A, Beland-Bonenfant S, Gascon G, Sutherland M, Lukaszewski MA, Cloutier A, Paradis P, Schiffrin EL and Nuyt AM. Activation of the Cardiac Renin-Angiotensin System in High Oxygen-Exposed Newborn Rats: Angiotensin Receptor Blockade Prevents the Developmental Programming of Cardiac Dysfunction. *Hypertension*. 2016;67:774-82.
2. Bertagnolli M, Huyard F, Cloutier A, Anstey Z, Huot-Marchand JE, Fallaha C, Paradis P, Schiffrin EL, Deblois D and Nuyt AM. Transient neonatal high oxygen exposure leads to early adult cardiac dysfunction, remodeling, and activation of the renin-angiotensin system. *Hypertension*. 2014;63:143-50.

SUPPLEMENTAL TABLES

Supplemental Table S1 – Echocardiographic data of neonatal P10 rats exposed to high oxygen (O_2) or kept in room air treated with vehicle cyclodextrin (CD), or the inclusion compounds of CD-Angio-(1-7) and CD-Ala.

Parameters	Room air			High O_2		
	CD (n=5)	CD-Angio-(1-7) (n=5)	CD-Ala (n=5)	CD (n=5)	CD-Angio-(1-7) (n=5)	CD-Ala (n=5)
HR (bpm)	266±11	246±18	279±8	270±23	257±20	261±17
LV M-mode						
IVSd (mm)	0.88±0.08	0.81±0.12	0.88±0.08	0.78±0.08	0.77±0.16	0.86±0.06
LVIDd (mm)	3.80±0.31	3.77±0.25	3.68±0.16	3.69±0.16	**3.31±0.21*†**	3.50±0.27
LVPWd (mm)	0.89±0.09	0.89±0.05	0.89±0.04	**0.72±0.12***	0.77±0.14	**0.72±0.10***
LV mass (mg)	99±11	93±4	95±9	**76±15***	**67±15***	**75±14***
LV mass index (mg/g)	4.5±0.6	4.0±0.2	4.3±0.3	4.1±0.6	3.8±0.9	4.0±0.9
LV systolic function						
LV Vol diastole (µL)	62±12	61±10	58±6	58±6	**45±7***	57±14
LV Vol systole (µL)	20±10	19±9	24±19	12±5	8±2	12±6
SV (µL)	43±5	46±8	45±17	48±5	38±6	42±5
FS (%)	39±10	39±11	41±9	47±7	**49±4***	46±5
Radial endocardial strain (%)	44±9	34±12	40±6	52±13	**71±20***	**34±12#**
Radial endocardial strain rate (1/s)	5.8±1.1	4.8±1.0	5.2±0.7	6.7±1.5	**8.9±2.0*†**	**6.4±2.0#**
LV diastolic function						
E velocity (mm/s)	652±113	701±141	707±62	**826±92***	**648±62†**	**839±144*#**
A velocity (mm/s)	483±72	484±65	469±75	535±158	453±93	512±98
E/A ratio	1.44±0.28	1.45±0.21	1.53±0.15	1.69±0.68	1.49±0.37	1.66±0.29
TD E' velocity (mm/s)	-17.4±3.4	-16.6±1.4	**-20.9±2.2#**	-20.2±2.0	-20.3±2.4	-21.5±2.4
E/E' ratio	38.4±8.5	42.6±9.7	34.1±3.6	41.2±5.4	**32.1±2.8†**	39.6±10.1
LV MPI	0.62±0.15	0.69±0.08	0.68±0.20	0.73±0.14	**0.56±0.06†**	0.66±0.20

Data presented as the mean ± SD. HR, heart rate; LV, left ventricle; IVSd, interventricular septal thickness in diastole; LVIDd, LV internal diameter in diastole; LVPWd, LV posterior wall thickness in diastole; SV, stroke volume; FS, fractional shortening; E, mitral E wave; A, mitral A wave; TD E', tissue Doppler E' wave; LV MPI, LV myocardial performance index. *$P<0.05$ versus room air treated with CD group, †$P<0.05$ versus high O_2-exposed group treated with vehicle CD, #$P<0.05$ versus CD-Ang-(1-7) treatment of respective exposure group. Two-way ANOVA and Bonferroni *post-hoc* test.

Supplemental Table S2 - Echocardiographic data of juvenile P28 rats exposed to high oxygen (O_2) or kept in room air treated with vehicle cyclodextrin (CD), or the inclusion compounds of CD-Angio-(1-7) and CD-Ala.

Parameters	Room air			High O_2		
	CD (n=8)	CD-Angio-(1-7) (n=8)	CD-Ala (n=7)	CD (n=6)	CD-Angio-(1-7) (n=7)	CD-Ala (n=8)
Body weight (g)	117±4	119±3	116±3	111±7	110±7	117±05
HR (bpm)	375±9	383±7	378±7	396±10	384±9	387±9
LV M-mode						
IVSd (mm)	1.05±0.03	**1.25±0.06***	1.16±0.03	1.13±0.06	1.14±0.08	**1.24±0.02***
LVIDd (mm)	5.45±0.13	5.35±0.19	5.32±0.11	5.46±0.19	5.34±0.20	5.52±0.24
LVPWd (mm)	1.18±0.07	1.24±0.05	1.29±0.07	1.16±0.08	1.22±0.08	1.28±0.07
LVIDs (mm)	3.48±0.17	3.41±0.14	**3.25±0.16**†	3.74±0.18	**3.38±0.23**†	3.52±0.30
FS (%)	36±2	36±1	39±2	**32±2***	**37±3**†	**37±4**†
LV mass (mg)	308±19	347±21	336±18	319±14	321±18	**350±19***
LV mass index (mg/g)	2.1±0.01	2.3±0.1	2.3±0.1	2.3±0.2	2.4±0.2	2.5±0.1
LV mitral valve						
E velocity (cm/s)	1.26±0.10	1.12±0.08	1.17±0.06	1.23±0.13	1.14±0.09	1.22±0.07
A velocity (cm/s)	0.89±0.07	0.91±0.07	0.80±0.06	0.87±0.08	0.92±0.04	0.90±0.05
E/A	1.43±0.07	1.28±0.12	1.48±0.18	1.42±0.08	1.27±0.12	1.35±0.04
Deceleration time (ms)	44±4	41±3	43±9	41±4	43±3	39±5

Data presented as the mean ± SD. HR, heart rate; IVSd interventricular septal thickness in diastole; LV, left ventricle; LVIDd, LV internal diameter in diastole; LVPWd, LV posterior wall thickness in diastole; LVIDs, LV internal diameter in systole; FS, fractional shortening; E, mitral E wave; A, mitral A wave. *P<0.05 versus CD room air group and †P<0.05 versus CD O_2-exposed group, two-way ANOVA and Bonferroni *post-hoc* test.

SUPPLEMENTAL FIGURES

Supplemental Figure S1

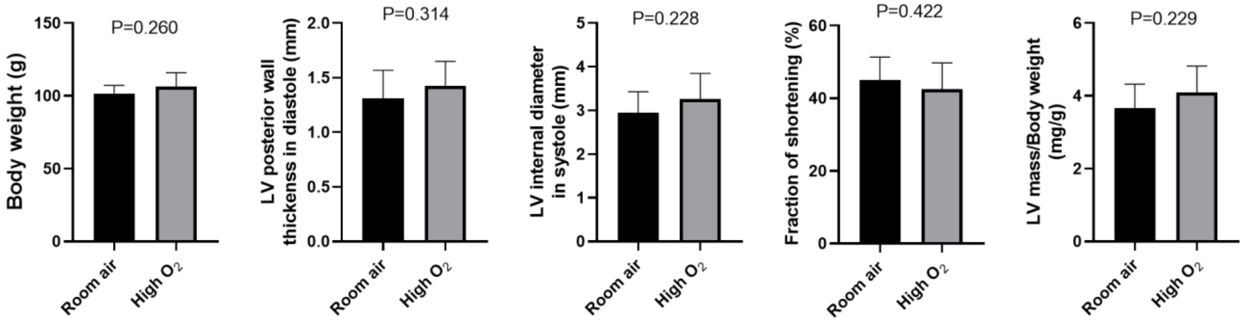

Supplemental Figure S1 – Echocardiography and heart morphometry analyses of female juvenile rats (P28) kept in room air or exposed to high O_2 from P3-P10 (n=9/group). Images were acquired using an ACUSON CV70 ultrasound imaging system (Siemens Medical Solutions, Burlington, ON) equipped with a 12 MHz scan head. LV, left ventricle. Unpaired Student t test.

Supplemental Figure S2

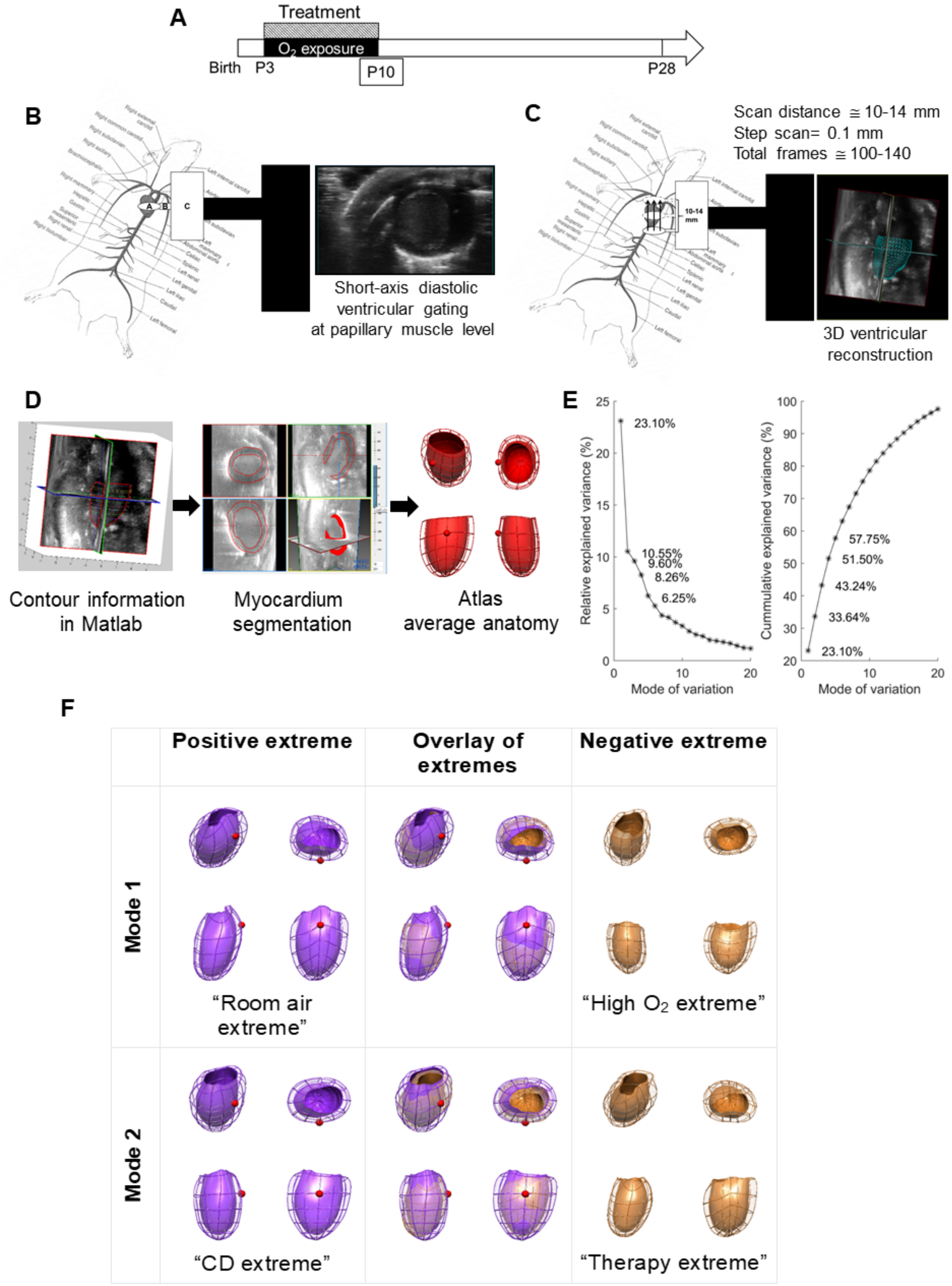

Supplemental Figure S2 – Neonatal echocardiography imaging and ventricular computational geometrical analyses. A) Study design demonstrating echocardiography imaging recorded at day 10 of life (P10) after 7 days of exposure (high-O_2 vs. room air) and treatment

(vehicle cyclodextrin (CD) versus CD-Angio-(1-7) or CD-Ala) interventions in neonatal rats. B) Schematic illustration of the imaging setting and acquisition using a VEVO3100 system (VisualSonics). C) Representative examples of the 3-dimensional (3D) volumes in VEVOLab software, of the ventricular segmentation using MITK software, and of the generated 3D ventricular meshes. D) Illustration of the two first principal component analysis (PCA) modes of anatomical variation identified in the 24 anatomies – each mode is represented by the two extremes of the linear geometrical transformation (orange and purple), with a red sphere pointing in the direction of the left ventricle lateral wall. E) Amount of variance explained by each mode (left) or cumulative variance explained by groups of modes (right). F) Illustration of the degrees of variation in mode 1 between room air controls and high O_2-exposed neonatal rats and in mode 2 between vehicle-treated (CD) and therapies.

Supplemental Figure S3

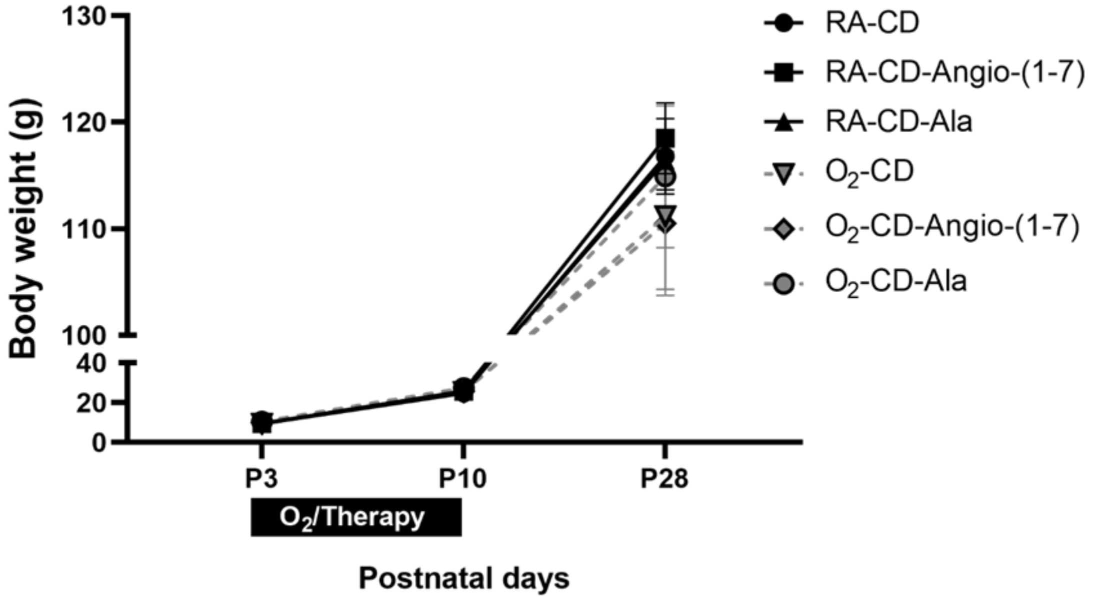

Supplemental Figure S3 – Growth curve from day 3 (P3) until day 28 (P28) of life. Weight of rats exposed to high oxygen (O_2) or kept at room air (RA) and treated with vehicle cyclodextrin (CD), or CD-Angio-(1-7) or with CD- Ala from P3-P10, and then followed until P28. No difference was observed between groups (n=6/group). Data presented as mean ± SEM. Two-way ANOVA for repeated measures.

Supplemental Figure S4

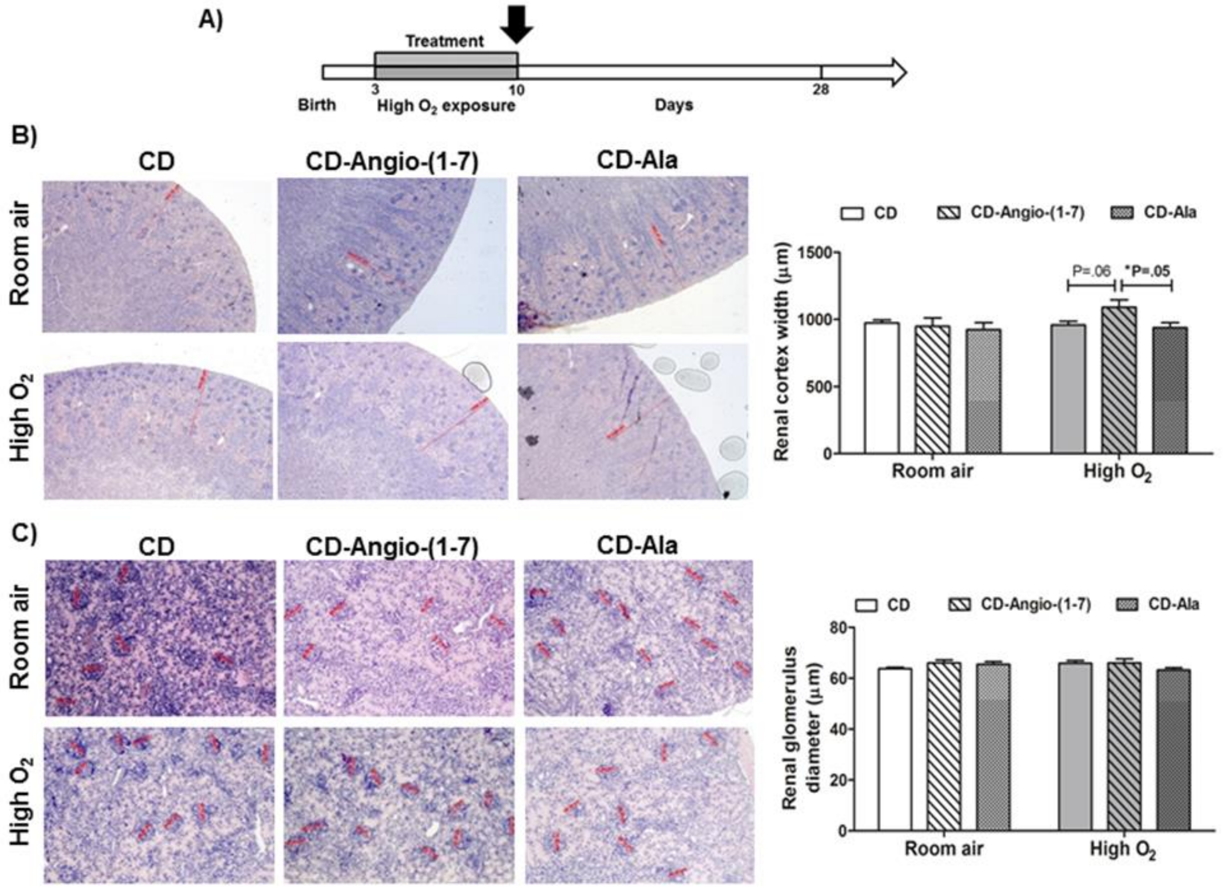

Supplemental Figure S4 – Effect of therapies on neonatal kidney maturation. A) Study design demonstrating that kidneys were collected at day 10 of life (P10) after 7 days of exposure and treatment interventions in neonatal rats. Representative histological images and quantification of renal B) cortex width and C) glomerulus diameter in rats (n=6/group) exposed to high oxygen (O_2) or kept at room air and treated with vehicle cyclodextrin (CD) or included CD-Angio-(1-7) or CD-Ala. Data presented as mean ± SEM. Two-way ANOVA followed by Bonferroni posthoc test. *$P<0.05$ vs. group indicated.
