# Supplementary material for: Reshaping the Preterm Heart: Shifting Cardiac Renin-Angiotensin System Towards Cardioprotection in Rats Exposed to Neonatal High-Oxygen Stress
Source: Hypertension. 2022 May 19;79(8):1789–803. doi: 10.1161/HYPERTENSIONAHA.122.19115 (PMC9278707; doi:10.1161/HYPERTENSIONAHA.122.19115)
Supplement: Supplementary file 2 [file hyp-79-1789-s002.pdf]

*** Short In Vivo Checklist**

*AHA - Preclinical Animal Testing : Prevention of bias is important for experimental cardiovascular research.* ***This short checklist must be completed, and the answers should be clearly presented in the manuscript as well.*** *The checklist will be used by reviewers and editors but will not be published. If a revision is requested, you will be required to complete at revision submission a more detailed checklist that will be published with the accepted article.*

*This study involves animals:*
Yes

**Animals**

Species, age, sex, strains, and sources of animals are described: Yes

**Randomization**

Randomization and allocation concealment were performed: Yes

**Blinding**

Blinding was performed: Yes

**Inclusions and Exclusions (a)**

Specific criteria for inclusions and exclusions are specified: Yes

**Inclusions and Exclusions (b)**

Criteria for inclusions and exclusions were set before the study: Yes

**Reporting of Excluded Animals**

All animals excluded after the randomization are reported: Yes

**Statistical Methods**

Statistical Methods are described: Yes

---

Date completed: 04/29/2022 17:08:45
User pid: 17794
